# Bone marrow mesenchymal stem cells promote remyelination in spinal cord by driving oligodendrocyte progenitor cell differentiation via TNFα/RelB-Hes1 pathway: a rat model study of 2,5-hexanedione-induced neurotoxicity

**DOI:** 10.1186/s13287-021-02518-z

**Published:** 2021-08-04

**Authors:** Shuangyue Li, Huai Guan, Yan Zhang, Sheng Li, Kaixin Li, Shuhai Hu, Enjun Zuo, Cong Zhang, Xin Zhang, Guanyu Gong, Ruoyu Wang, Fengyuan Piao

**Affiliations:** 1 School of Public Health, Dalian Medical University, Dalian, Liaoning 116044 People’s Republic of China; 2 Department of Obstetrics and Gynecology, No. 967 Hospital of the Joint Logistics Support Force of the Chinese PLA, Dalian, People’s Republic of China; 3 Xunyi Center for Disease Control and Prevention, Xunyi, Shanxi, 711300 People’s Republic of China; 4 Department of Biochemistry, Dalian Medical University, Dalian, Liaoning, 116044 People’s Republic of China; 5 Xian Center for Disease Control and Prevention, Xian, 710054 People’s Republic of China; 6 College of Stomatology, Dalian Medical University, Dalian, Liaoning 116044 People’s Republic of China; 7 Department of Clinical Nutrition, The First Affiliated Hospital of Dalian Medical University, Dalian, Liaoning 116011 People’s Republic of China; 8 Integrative Laboratory, Affiliated Zhongshan Hospital of Dalian University, Dalian, 116001 People’s Republic of China; 9 Department of Medical Oncology, Affiliated Zhongshan Hospital of Dalian University, Dalian, People’s Republic of China

**Keywords:** Bone marrow-mesenchymal stem cells, 2,5-Hexanedione, Demyelination, Remyelination, Oligodendrocyte precursor cells, Notch1, TNFα, RelB, Hes1, NGF

## Abstract

**Background:**

*N*-hexane, with its metabolite 2,5-hexanedine (HD), is an industrial hazardous material. Chronic hexane exposure causes segmental demyelination in the peripheral nerves, and high-dose intoxication may also affect central nervous system. Demyelinating conditions are difficult to treat and stem cell therapy using bone marrow mesenchymal stem cells (BMSCs) is a promising novel strategy. Our previous study found that BMSCs promoted motor function recovery in rats modeling hexane neurotoxicity. This work aimed to explore the underlying mechanisms and focused on the changes in spinal cord.

**Methods:**

Sprague Dawley rats were intoxicated with HD (400 mg/kg/day, i.p, for 5 weeks). A bolus of BMSCs (5 × 10^7^ cells/kg) was injected via tail vein. Demyelination and remyelination of the spinal cord before and after BMSC treatment were examined microscopically. Cultured oligodendrocyte progenitor cells (OPCs) were incubated with HD ± BMSC-derived conditional medium (BMSC-CM). OPC differentiation was studied by immunostaining and morphometric analysis. The expressional changes of Hes1, a transcription factor negatively regulating OPC-differentiation, were studied. The upstream Notch1 and TNFα/RelB pathways were studied, and some key signaling molecules were measured. The correlation between neurotrophin NGF and TNFα was also investigated. Statistical significance was evaluated using one-way ANOVA and performed using SPSS 13.0.

**Results:**

The demyelinating damage by HD and remyelination by BMSCs were evidenced by electron microscopy, LFB staining and NG2/MBP immunohistochemistry. In vitro cultured OPCs showed more differentiation after incubation with BMSC-CM. Hes1 expression was found to be significantly increased by HD and decreased by BMSC or BMSC-CM. The change of Hes1 was found, however, independent of Notch1 activation, but dependent on TNFα/RelB signaling. HD was found to increase TNFα, RelB and Hes1 expression, and BMSCs were found to have the opposite effect. Addition of recombinant TNFα to OPCs or RelB overexpression similarly caused upregulation of Hes1 expression. The secretion of NGF by BMSC and activation of NGF receptor was found important for suppression of TNFα production in OPCs.

**Conclusions:**

Our findings demonstrated that BMSCs promote remyelination in the spinal cord of HD-exposed rats via TNFα/RelB-Hes1 pathway, providing novel insights for evaluating and further exploring the therapeutical effect of BMSCs on demyelinating neurodegenerative disease.

**Supplementary Information:**

The online version contains supplementary material available at 10.1186/s13287-021-02518-z.

## Background

*N*-hexane, an organic solvent widely used in industry, is a neurological hazardous material affecting health of millions of workers in developing countries [1]. The neurotoxic effects of n-hexane are mediated by its reactive metabolite 2,5-hexanedione (HD). Chronic exposure to *n*-hexane primarily causes sensorimotor polyneuropathy in the peripheral nervous system (PNS), with clinical presentation including muscle wasting, weakness and sensory impairment [2–4]. Pathophysiologically, *n*-hexane/HD neurotoxicity has been suggested to cause segmental demyelination in peripheral nerve fibers [5, 6]. In addition, some evidences showed that hexane may also cause damage to the central nervous system (CNS). Earlier studies described persistent residual spasticity and hyperactive tendon reflexes (descending motor tracts) and ataxia (spinocerebellar tracts) occurred in hexane-intoxicated patients after recovery from PNS symptoms [7, 8]. The review by Huang CC also documented hexane-associated CNS symptoms such as cephalalgia, sleep problem and gait spasticity [4]. In experimental animals, high-dose HD intoxication was found to cause demyelination in PNS and spinal cords and has been used to study the mechanisms of HD neurotoxicity and evaluate the therapeutical potential of various reagents for demyelinating conditions [9].

Myelination of nerve fibers comprises multiple oligodendrocytes (OLs), as myelin sheaths, segmentally wrapping around an axon. Appropriate myelination permits accelerated conduction of electrical impulses, whereas loss of myelin sheaths (loss of oligodendrocytes, i.e. demyelination) decelerates conduction. Moreover, various studies have shown that persistent demyelination may result in axonal damage and neuron degeneration [10, 11]. Indeed, therapeutical approach promoting timely recovery of damaged myelin sheath is an urgent medical need for many neurological disorders, but to the best of our knowledge, progression has been limited.

Stem cell therapy using bone marrow mesenchymal stem cells (BMSCs) is a promising therapeutical advance in the recent decade. Several studies have shown that BMSCs might exert certain functions on the oligodendrocyte precursor cells (OPCs), the progenitor cells of mature oligodendrocytes (OLs), and promote their enrichment to the sites of injury, differentiation and remyelination of the damaged nerve fibers [12–14]. For example, in vitro co-culture of OPCs with BMSCs has been found to preferentially drive the differentiation of OPCs into oligodendrocytes (oligodendroglial fate) rather than the astrocytes (astroglial fate) [15, 16]. In a streptozotocin (STZ)-treated rat model of diabetes-induced sciatic nerve demyelination, injection of BMSCs into hind limb was found to promote remyelination [17]. In another rat model of cuprizone-induced demyelination, BMSCs transplantation was found to target on oligodendrocyte progenitors and promote remyelination [18]. These findings inspired our interest to evaluate the therapeutical potential of BMSC transplantation in *n*-hexane/HD-induced demyelinating neurotoxicity.

Further investigations remain needed to decipher the underlying mechanisms between BMSC and OPC interaction. Existing publications have suggested that Notch signaling pathway plays a pivotal role in the maintenance, proliferation and differentiation of OPCs [19, 20]. Deletion of Notch1 gene has been found to enhance oligodendrocyte differentiation and encourage remyelination after cuprizone-induced injury [21]. Hes1 is the main effector of Notch signaling pathway. It has been reported that Hes1 negatively regulates OPCs differentiation [22]. However, another study by Mathieu et al. reported that Notch signaling activated by F3/contactin positively promoted OL mature in brain and neurosphere culture [23]. Hence, the exact role of Notch and Hes1 signaling in the process of remyelination necessitates further clarification.

In the present study, we modeled HD-induced neurotoxicity in adult rats, administrated BMSCs via tail vein injection and intensively analyzed the demyelination and remyelination changes in spinal cord. BMSCs were found to significantly recover the HD-associated demyelinating damage. Mechanistically, the neuroprotective effect of BMSCs was associated with nerve growth factor (NGF) and TNFα/RelB-dependent negative regulation of Hes1 expression independent of Notch1 pathway. Our study provided novel insight for evaluation of the therapeutic potential of BMSCs on demyelinating nerve injury.

## Methods

### Animal experiments

The animal experiments in the present study were approved (with license number: AEE19095) by the Ethical Committee of Dalian Medical University, China, and performed in accordance with the Animal Handling Guideline of Dalian Medical University. As being described in our earlier study [24], Sprague Dawley (SD) male rats (9 weeks old) were intoxicated with HD by intraperitoneal injection (i.p.) at dosages of 400 mg/kg/day (five times per week). The untreated control group animals received an equivalent volume of 0.9% saline by i.p. On completion of 5 weeks of HD administration, rats were transplanted with a bolus injection of BMSCs (5 × 10^7^ cells/kg) via tail vein and observed for 5 weeks until necropsy.

### BMSCs isolation and culture

The isolation and culture of BMSCs from adult SD rats were performed as being described in our previous study [25]. Briefly, bone marrow was excised, dissociated into single cell suspension and cultured in Dulbecco’s modified eagle medium–low glucose (DMEM-LG, Gibco, USA) supplemented with 10% fetal bovine serum (FBS, HyClone, USA), 100 U/ml penicillin and 100 μg/ml streptomycin (Beyotime, China). Non-adherent cells were removed after 24 h by replacing the media. The adherent population was cultured and those cells between passages 3 and 5 were used for further in vitro experiments. The biological features of these BMSCs were as being characterized in our previous publication [26].

### BMSC-CM (BMSC-derived conditioned medium) preparation

BMSC-CM (BMSC-derived conditioned medium) was prepared according to our earlier publications [25]. BMSCs were subcultured for 3–5 passages, washed with PBS and added with fresh DMEM with 10% FBS for 24 h. The culture medium was collected, centrifuged at 1500 rpm (200 g) for 10 min at 4 °C and transferred to a centrifugal column with a 3 kDa cutoff (Millipore, Billerica, MA, USA). The column was centrifuged at 3500 rpm (3500 g) for 45 min at 4 °C. The pass-through was desalted according to the manufacturer’s instruction and sterilized by filtration with 0.22 μm membrane.

### OPCs culture and treatment

Primary OPCs were harvested and cultured following the protocol of McCarthy et al. [27]. Brain cortices of P1–P2 rat pups were dissected, dissociated into single cell suspension with a mixed population of astrocytes, oligodendrocytes and microglials, plated on flasks pre-coated with 20 μg/ml poly-D-lysine (PDL) and cultured in DMEM/F12 medium (Gibco) supplemented with 10% FBS, 1% MEM nonessential amino acids (MEM NEAA, Gibco), 1% L-glutamine (Gibco) and 1% Pen/Strep (Gibco). After 10 days of growth, the cultured cells formed a bedding layer of mixed glial cells and a phase-dark, process-bearing OPCs on top of the bedding layer. In order to grossly remove the microglial cells, the flask was shaken at 200 rpm for 2 h at 37 °C, and then, the culture medium was removed. Next, ten milliliters of fresh medium were added and the flask was shaken at 250 rpm for additional 20 h. The cell suspension containing the OPCs, astrocytes and some remaining microglials was transferred to an untreated Petri dish. Taking advantage of the difference in attaching ability between OPCs and astrocytes/microglials, the flask was briefly incubated for 30–60 min in incubator, allowing the cells other than OPCs to seed. Then, the cell suspension was centrifuged at 100 g (800 r.p.m.) for 10 min, plated at a density of 8 × 10^4^ cells/cm^2^ in PDL-precoated flasks for 5 h, switched to OPC basal medium (Neurobasal medium supplemented with 2% B27 (Gibco), 10 ng/ml PDGFaa (PeproTech), 1%L-glutamine (Sigma) and 1% Pen/Strep (Gibco)) and cultured for 3 days. To induce neurotoxicities, cells were treated with HD (25 mM) or saline for 24 h and then were treated with BMSC-CM (15%, v/v) and with or without additional NGF neutralizing antibody (10 μM), NGF (100 μM), or K252a (0.1 μM) (for k252a, 1 h-treatment followed by replacing with k252a-free culture medium, as being described elsewhere [28] for additional 24 h.

### Electron microscope analysis

Fixed spinal cord was gradient-dehydrated, embedded and then sliced with an ultramicrotome. These sections were stained with uranyl acetate and lead citrate. Transmission electron microscope (TEM, H/7500, Hitachi, Japan) was utilized to observe the pathological changes of the myelin.

### Luxol-fast blue (LFB) staining

Spinal cord sections were immersed in ethanol/chloroform solution (1:1, v/v) for 5 min and 95% ethanol for another 5 min. Sections were stained with 0.1% LFB (Sigma, USA) solution overnight and immersed in cresol purple for 4 min. Then, sections were washed with 70% ethanol and dehydrated in 99% ethanol.

### Western blot

Fresh tissues or cultured cells were homogenized in ice-cold RIPA buffer (Beyotime, China) supplemented with 1% proteinase inhibitor and phosphatase inhibitor. The proteins were separated on SDS-PAGE and then transferred to PVDF membrane (Millipore, France). The membrane was incubated with the following antibodies: MBP (1:1000, Abcam, USA), Hes1 (1:1000, Sigma, USA), Jagged1 (1:500, Abcam, USA), Notch1 (1:1000, Abcam, USA), NICD (1:500, Cell Signing Technology, USA), RBPJκ (1:1000, Abcam, USA), TNFα (1:500, Abcam, USA), RelB (1:2000, Abcam, USA) and β-actin (1:500, ZS-Bio, China). The membrane was incubated with horseradish peroxidase-conjugated second antibody (1:5000, Sigma, USA) and visualized with enhanced chemiluminescence (Beyotime, China). Quantification was performed using UVP BioSpectrum Multispectral Imaging System (Ultra-Violet Products Ltd. USA), and quantification results were shown in Additional file 1: Figure 1.

### Immunofluorescence

The frozen Sects. (10 μM) of spinal cords or cells were blocked and then incubated with anti-NG2 (1:300, Abcam, USA) antibody overnight at 4 °C, washed with PBS and incubated with Alexa-488 (green)-conjugated secondary antibody. The sections were next incubated with anti-MBP antibody (1:200, Abcam, USA) overnight at 4 °C, washed, incubated with Alexa-594 (red) conjugated secondary antibody, counterstained using DAPI and imaged under a confocal microscope.

### Morphometric analysis of immunofluorescent images

For analysis of immunostained tissue sections, three independent sections per animal were stained and ten images per section were captured for morphometric analysis. The images were analyzed using ImageJ software (NIH, US) and fluorescent intensity was evaluated as the integrated optical density (IOD) per image field and averaged over all the images being processed for each animal. Alternatively, for analysis of immunostained cultured cells, three coverslips grown with cells per treatment group were used and ten images per coverslip were captured for morphometric analysis using ImageJ. The total number of cells and the number of MBP-positive cells were estimated using the number of DAPI-positive areas and the number of MBP-staining positive areas per image, respectively. The percentage of MBP-positive cells was expressed as number of MBP-positive area/number of DAPI-positive area and averaged over all the images per treatment group.

### Real-time PCR

Total RNA was extracted from the spinal cords using RNAiso Plus (Takara, Tokyo, Japan). RNA was reverse transcribed using a Reverse Transcription Kit (Takara, Tokyo, Japan). Real-time q-PCR was performed using a SYBR Green PCR kit (Takara, Tokyo, Japan) using the TP800 Real-Time PCR Detection System (Takara, Tokyo, Japan). The following primers were used: Hes1, 5′-CAT TGG CTG AAA GTT ACT GTG G-3′/5′-TTG AGG GTT TAT TAT GTC TTA GGG-3′; Jagged1, 5′-TGC GAC CAG AAT GGC AAC A-3′/5′-GTC ACC TGG GAG TTT GCA GGA-3′; Notch1, 5′-CCC ATT ACA TGC CGC TGT TTC-3′/5′-CAT CAT GCA TTC GGG CATC-3′; NICD, 5′-ACG CTG CTG TTG TGC TCC T-3′/5′-GGC AAT CGG TCC ATG TGA T-3′; RBPJ, 5′-TTG CTT ACC TTC AGG CGT GTG-3′/5′-GCC CAA TGA GTC TGC TGC AA-3′; TNF, 5′-TCA GTT CCA TGG CCC AGA C-3′/5′-GTT GTC TTT GAG ATC CAT GCC ATT-3′; RelB, 5′-CCT CTG AGC TGC GGA TTT G-3′/5′-GTC CTC GTA TGG TGG CGT TT-3′; GAPDH, 5′-GGC ACA GTC AAG GCT GAG AAT G-3′/5′-ATG GTG GTG AAG ACG CCA GTA-3′. The PCR programs were 95 °C for 30 s, 55 °C for 30 s and 72 °C for 30 s for 40 cycles. Data were normalized to β-actin.

### Plasmids and lentivirus construction

The plasmid pWPXL-Hes1 and pCDH-CMV-MCS-EF1α-puro lentiviral vectors were obtained from Addgene (USA). Hes1 amplification product was cloned into through Xho1 and EcoR1 sites. The resulting lentivirus was subsequently used to infect OPCs.

### Statistical analysis

All results were expressed as mean ± S.D. The statistical significance was evaluated using one-way analysis of variance (ANOVA), followed by LSD test and performed using SPSS 13.0 statistical software. The Shapiro–Wilk normality test was used to analyze data for Gaussian distribution and homogeneity of variances using SPSS. The p values less than 0.05 were considered to be significant.

## Results

### Effect of BMSCs on remyelination in the spinal cord of HD-exposed rats

**Fig. 1:**
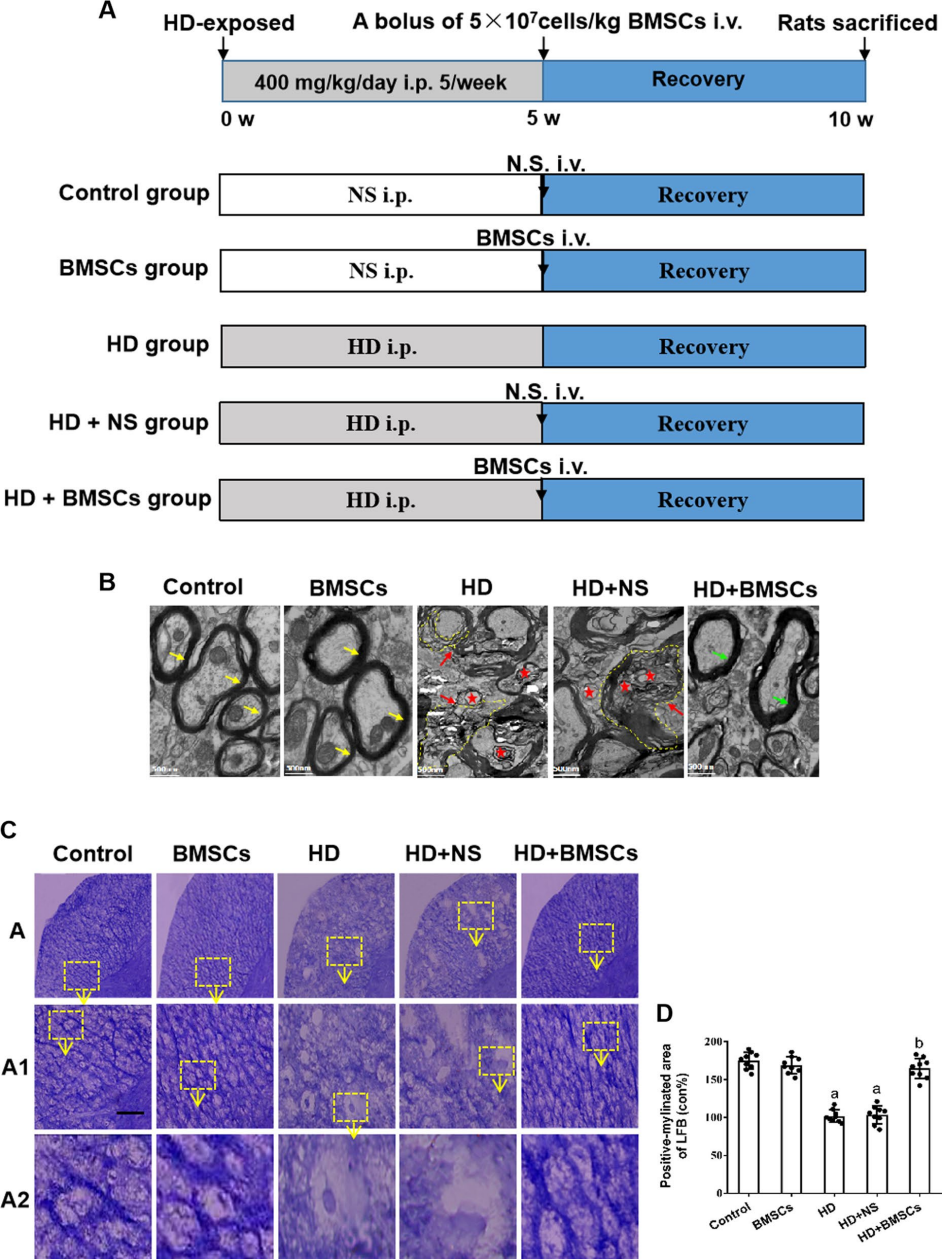
Changes of myelin sheath in the spinal cord of HD-exposed rats with or without BMSCs. **a** Schematic diagram of experimental design. SD rats were intraperitoneally injected with HD (400 mg/kg in saline) or saline for 5 consecutive weeks (5 times per week) to induce demyelinating damage. Then, rats were grafted with 5 × 10^7^/kg body weight BMSCs by tail vein injection and observed for another 5 weeks for remyelinating recovery. Control group: rats received normal saline (NS) i.p. injection and NS i.v. injection; BMSCs group: rats received normal saline (NS) i.p. injection and BMSCs i.v. injection; HD group: rats received HD i.p. injection and no further treatment; HD + NS group: rats received HD i.p. injection and NS i.v. injection; HD + BMSC group: rats received HD i.p. injection and BMSC i.v. injection; **b** representative electron-microscope images of spinal cord. Yellow arrows represent normal myelin, red arrows represent structurally deranged myelin, featuring with segmental loss and separation of myelin sheath and vacuole-like denaturation (red star), and Green arrows represent recovered myelin sheath structure following BMSCs treatment. **c** Luxol fast blue (LFB)-stained images. Scale bar, 50 μm. **d** Quantification of LFB^+^ area. Quantified data are shown as mean ± SD. a: Compared with control group, *p* < 0.05; b: compared with HD group, *p* < 0.05

Our earlier report observed that BMSCs transplantation alleviated motor neuron dysfunction and pathological damage in HD-intoxicated rats [29]. To investigate the underlying mechanism associated with this observation, the present study employed a SD rat model administrated with HD (400 mg/kg/day, i.p) for 5 weeks (intoxication period), then treated with either BMSCs transplantation (HD + BMSCs group, 5 × 10^7^ cells/kg suspended in saline as a bolus dose) or normal saline (HD + NS group, equal amount of saline used as control) by tail vein injection and observed for another 5 weeks (recovery period). Two groups of mice receiving no treatment (Control group) or only HD treatment (HD only group) were necropsied at the end of intoxication period, and three groups of mice receiving BMSC-only treatment, HD + BMSCs treatment and HD + saline treatment were necropsied at the end of recovery period. The animal experiment was designed as being diagramed in Fig. 1a.

In order to examine whether the demyelinating damage caused by HD had been recovered by BMSC transplantation, spinal cords were harvested and subjected to ultrastructure analysis using transmission electron microscopy. As shown in Fig. 1b, comparing to the control group or the BMSC group with intact myelinated nerves (yellow arrow), HD group and HD + NS group demonstrated similar demyelinating structure of spinal cord axons featuring large swollen axons surrounded with disintegrated, thinner and split myelin sheaths (red arrow). In contrast, remyelination was observed in HD + BMSCs group showing reconstructed structure with more compact and thicker myelin sheaths (green arrow). Furthermore, the changes of myelin structure were also assessed using Luxol-fast blue (LFB) staining (Fig. 1c, d), which is a frequently used procedure for the demonstration of myelin structure in nervous tissue. Comparing to the fainter and patchy depigmenting staining feature in the HD group and HD + NS group, HD + BMSCs group showed noticeably increased LFB staining with more refined substructure (Fig. 1c, d). Together, these results suggested that HD caused severe demyelination in rat spinal cords, spontaneous remyelination without therapeutical intervention played a limited role to repair the damage, but BMSC transplantation could markedly promote regrowth of myelin sheath upon the injured nerve fibers.

### BMSCs promote OPCs differentiation to mature OLs *in vivo* and *in vitro*

**Fig. 2:**
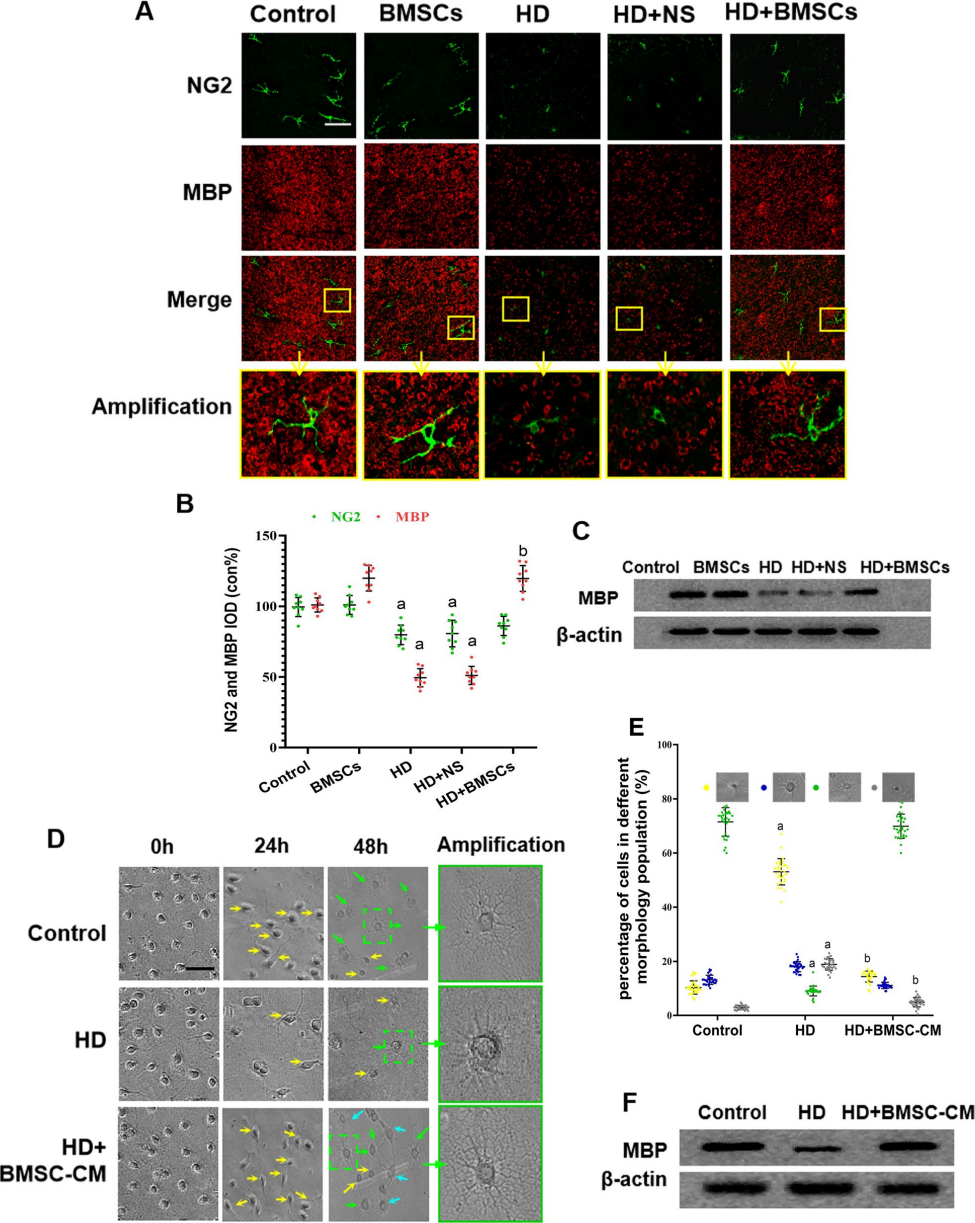
MBP expression and the number of OLs in the spinal cord of HD-exposed rats with or without BMSCs. **a** Representative images stained for NG2 (green, marker of OPCs) and MBP (red, marker of mature OLs). Scale bar, 50 μm. **b** Quantification of MBP^+^ and NG2^+^ cells. **c** Western blot results of MBP expression in the spinal cords of HD/BMSC-treated rats. **d** Representative phase images of cultured OPCs treated with HD/HD + BMSC-CM. Yellow arrows represent OPCs, cyan arrows represent immature OLs, and green arrows represent mature OLs. Scale bar, 50 μm. **e** Quantification of OPCs, immature OLs, mature OLs and death cell. **f** Western blot of MBP in HD/BMSC-treated OLs. Quantified data are shown as mean ± SD. a: Compared with control group, *p* < 0.05; b: compared with HD group, *p* < 0.05

Next, we sought to understand the associated mechanism(s) responsible for BMSC-promoted remyelination. Since it has been reported that the differentiation of OPCs to mature OLs is a rate-limiting step of remyelination [30], experiments were carried out to compare the number of immature OPCs and mature OLs in spinal cord sections from HD-intoxicated rats, using NG2 and MBP as immunoblotting markers, respectively. As shown in Fig. 2a, b, HD treatment was associated with significantly reduced number of NG2 as well as MBP positive cells, whereas BMSC grafting significantly recreated the number of myelinating oligodendrocytes in the spinal cord tissues. Such an observation was also confirmed by Western blot using whole tissue lysate of spinal cords (Fig. 2c). These results indicated that BMSCs exerted certain impact increasing the number of available OPCs and OLs in the spinal cords after HD-induced neurotoxicity in vivo.

To further determine the regulatory factors in BMSCs’ impact on oligodendrocyte differentiation, in vitro experiments were conducted to replicate the aforementioned observations using cultured primary cells. Cultured primary OPCs were treated with 25 mM HD or vehicle for 24 h and then incubated with BMSC-derived conditioned medium (BMSC-CM), the acellular medium harvested from BMSCs culture, for additional 2 days, as being described in the Materials & Methods section. The cells were subjected to morphometric analysis to distinguish and quantify the different oligodendrocyte population. Importantly, our in vitro results also found that BMSC-CM treatment significantly increased the number of differentiating immature OLs (cyan arrows) and mature OLs (green arrows), comparing with HD group (Fig. 2d, e). Elevated level of MBP protein was detected in the BMSC-CM-treated cells (Fig. 2f), suggesting that BMSCs might promote oligodendrocyte maturation in HD-intoxicated OPCs.

### BMSCs promote OPCs differentiation via downregulating Hes1

**Fig. 3:**
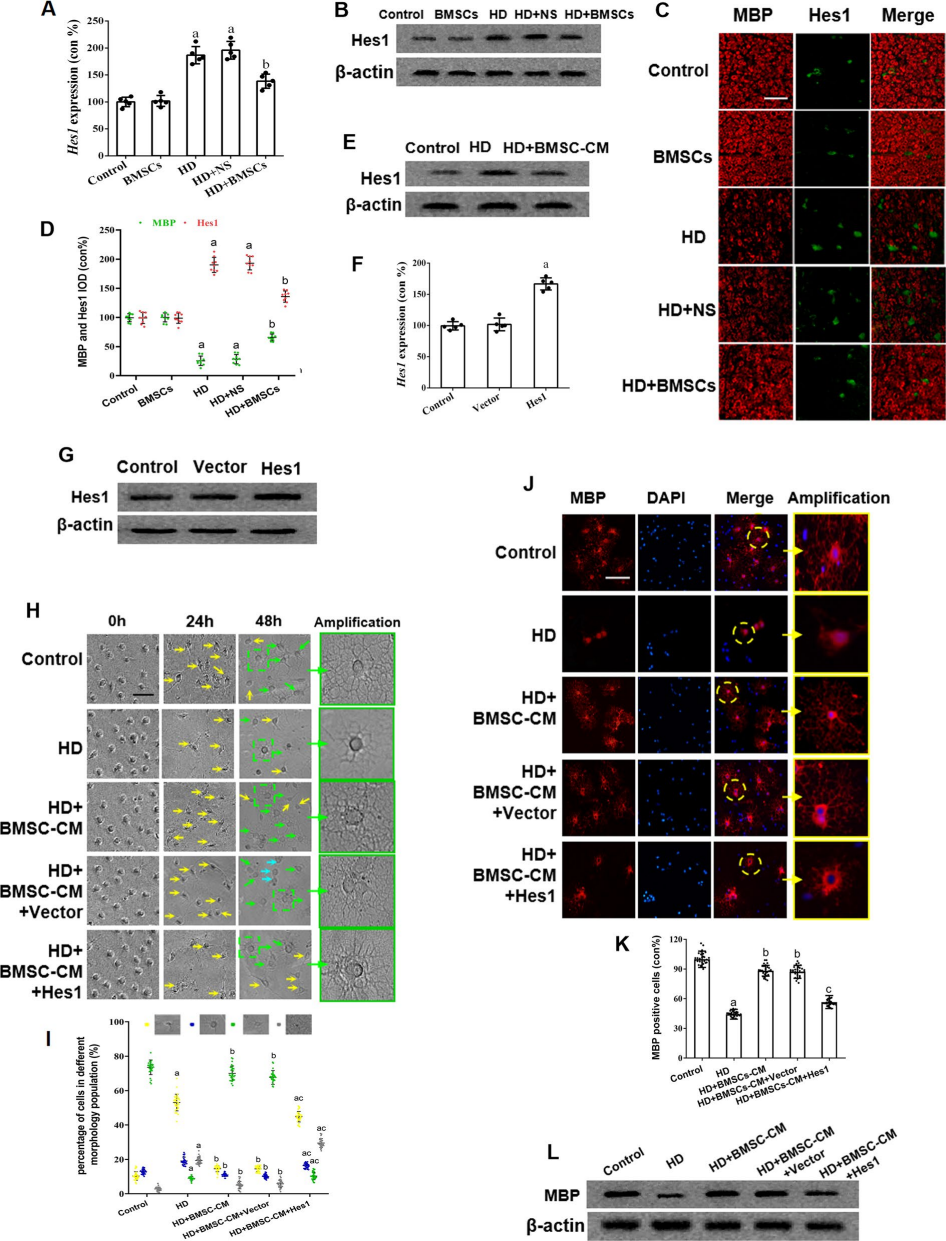
Hes1 expression and its relationship with the BMSCs-induced remyelination in HD-exposed rats. **a** qRT-PCR results showing Hes1 mRNA level in the spinal cords of HD/BMSCs-treated rats. **b** Western blot results showing Hes1 protein level in the spinal cords of HD/BMSC-treated rats. **c** Representative images showing MBP (red) and Hes1 (green) staining in the spinal cords of HD/BMSCs-treated rats. Scale bar, 50 μm. **d** Quantification of MBP^+^ and Hes1^+^ cells. **e** Western blot results showing Hes1 protein expression in cultured OPCs treated with HD/HD + BMSC-CM. **f, g** qRT-PCR and western blot results showing Hes1 mRNA and protein levels in Hes1-overexpressing OPCs but not vector-transfected OPCs. **h** Representative phase images of cultured OPCs treated with HD/HD + BMSC-CM/HD + BMSC-CM + vector/HD + BMSC-CM + Hes1 overexpression. Yellow arrows represent OPCs, green arrows represent mature OLs, and cyan arrows represent immature OLs. Scale bar, 50 μm. **i** Quantification of OPCs, immature OLs, mature OLs and death cell. **j** Representative images of MBP staining in the above described OPCs. Scale bar, 50 μm. **k** Quantification of MBP^+^ cells. **l** Western blot results showing MBP level. Quantified data are shown as mean ± SD. a: Compared with control group, *p* < 0.05; b: compared with HD group, *p* < 0.05; c: compared with HD + BMSCs group, *p* < 0.05

Some studies have reported the inhibitory effects of hairy-enhancer-of-split (Hes) family members, such as Hes1 that is highly expressed by OPCs, on oligodendrogenesis [30–32]. To determine whether Hes1 participated in the effect of BMSCs on OPC differentiation, the mRNA and protein levels of Hes1 in spinal cords were measured and compared among different HD/BMSCs treatment groups. The results showed that HD intoxication increased while BMSCs transplantation decreased mRNA and protein expression of Hes1, respectively (Fig. 3a, b). In line with this, immunostaining demonstrated significant elevation of Hes1 positive cells in the spinal cords of HD group rats and reduction of Hes1 positive cells in those rats grafted with BMSCs (Fig. 3c, d).

Similar to the in vivo findings, western blot analysis of the OPCs cultured in BMSC-CM also showed that Hes1 level was increased by HD and decreased by BMSC-CM treatment (Fig. 3e). Next, we overexpressed Hes1 gene in cultured OPCs (Hes1^+^ OPCs) and confirmed that the Hes1 mRNA and protein expression were higher (Fig. 3f, g, respectively). Meeting our expectation, as shown in Fig. 3h, i, morphological analysis revealed that in the Hes1-overexpressing OPCs, the beneficial effect of BMSC-CM was abolished, with more mature OLs (green arrows) presenting in HD-treated OPCs cultured in BMSC-CM but less mature OLs being identified under the condition that Hes1 was overexpressed. Consistent with this, immunostaining and western blot results (Fig. 3j–l, respectively) also revealed that the change of MBP in response to BMSC-CM was canceled after Hes1 overexpression. Together, these results indicated that BMSCs promoted OPC differentiation into mature OLs in a Hes1-dependent manner.

### BMSCs regulate Hes1 expression in OPCs independent of Notch1 signaling

**Fig. 4:**
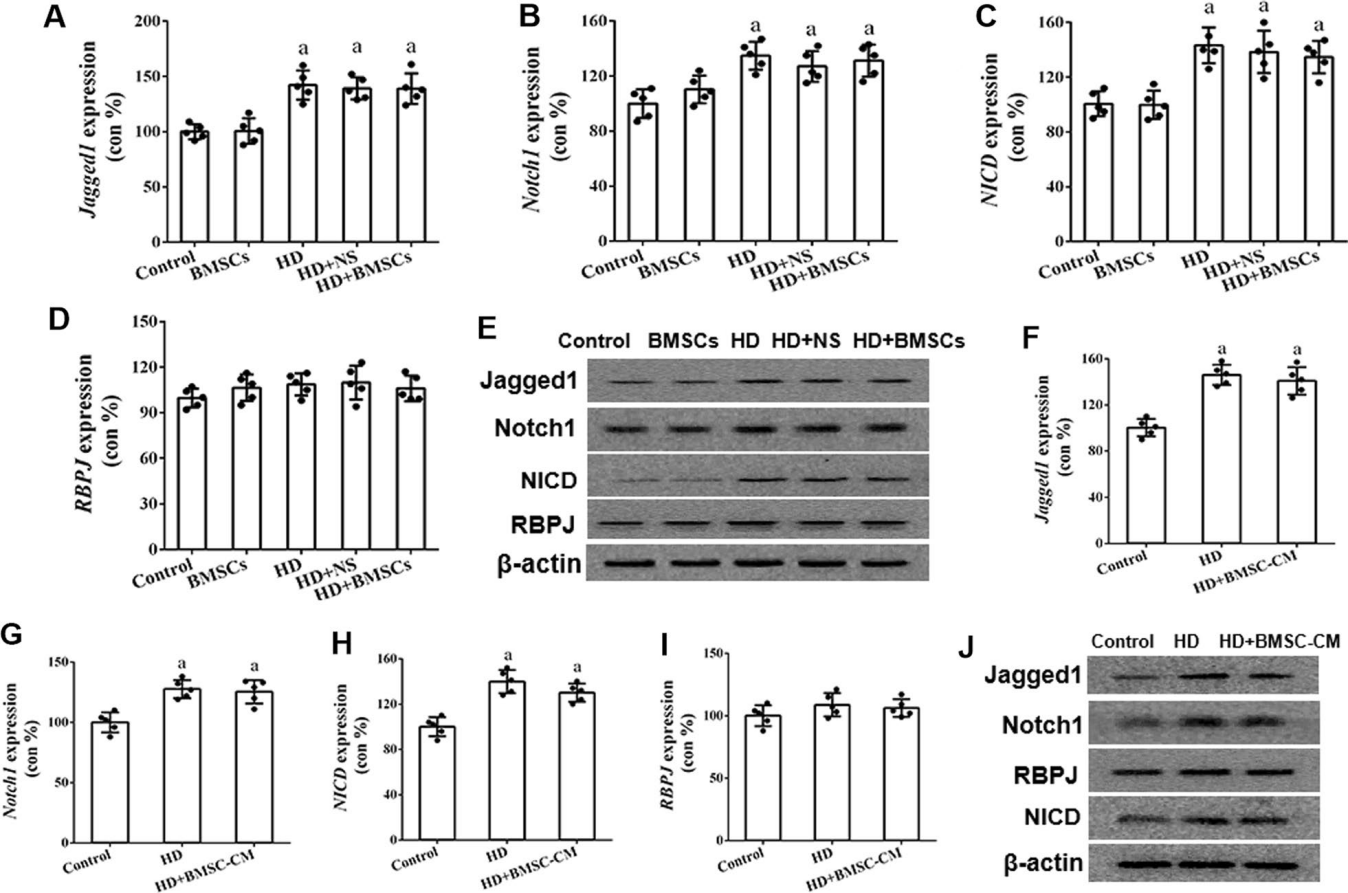
Expression of Jagged1, Notch1, NICD and RBPJ in the spinal cord of HD-treated rats and HD-treated OPCs with or without BMSCs/BMSC-CM. **a**–**d** qRT-PCR results showing Jagged1, Notch1, NICD and RBPJ mRNA expression in the spinal cords of HD/BMSC-treated rats. **e** Western blot results showing Jagged1, Notch1, NICD and RBPJ protein expression in the spinal cords of HD/BMSCs-treated rats. **f**–**i** qRT-PCR results of Jagged1, Notch1, NICD and RBPJ in HD/BMSC-CM-treated OPCs. **j** Western blot results of Jagged1, Notch1, NICD and RBPJ in HD/BMSC-CM-treated OPCs. Bar graph present mean ± SD. a: Compared with control group, *p* < 0.05; b: compared with HD group, *p* < 0.05; c: compared with HD + BMSCs/BMSC-CM group, *p* < 0.05

Earlier studies have implied that Hes1 expression is commonly regulated by Notch1 signaling in adult OPCs [33]. Therefore, our work continued by comparing the expressional changes of several key Notch1 signal transducers in the spinal cords of HD-intoxicated rats with or without BMSCs transplantation. As shown in Fig. 4, no significant difference on mRNA level and protein level was observed in *Jagged1*, *Notch1*, *NICD* or *RBPJ* (Fig. 4a–e) expressions in spinal cords comparing among the HD group, HD + NS group and HD + BMSC group. Further analyses were also carried out to detect these Notch1 players in HD-dosed OPCs ± BMSC-CM and failed to evidence any expressional difference in *Jagged1, Notch1*, *NICD* and *RBPJ* (Fig. 4f–j). Collectively, these in vivo and in vitro data strongly argued that in our scenario, Hes1 expression in OPCs was regulated by BMSCs in a Notch1-independent manner.

### The regulation of Hes1 by BMSCs in oligodendrogenesis is associated with TNFα

**Fig. 5:**
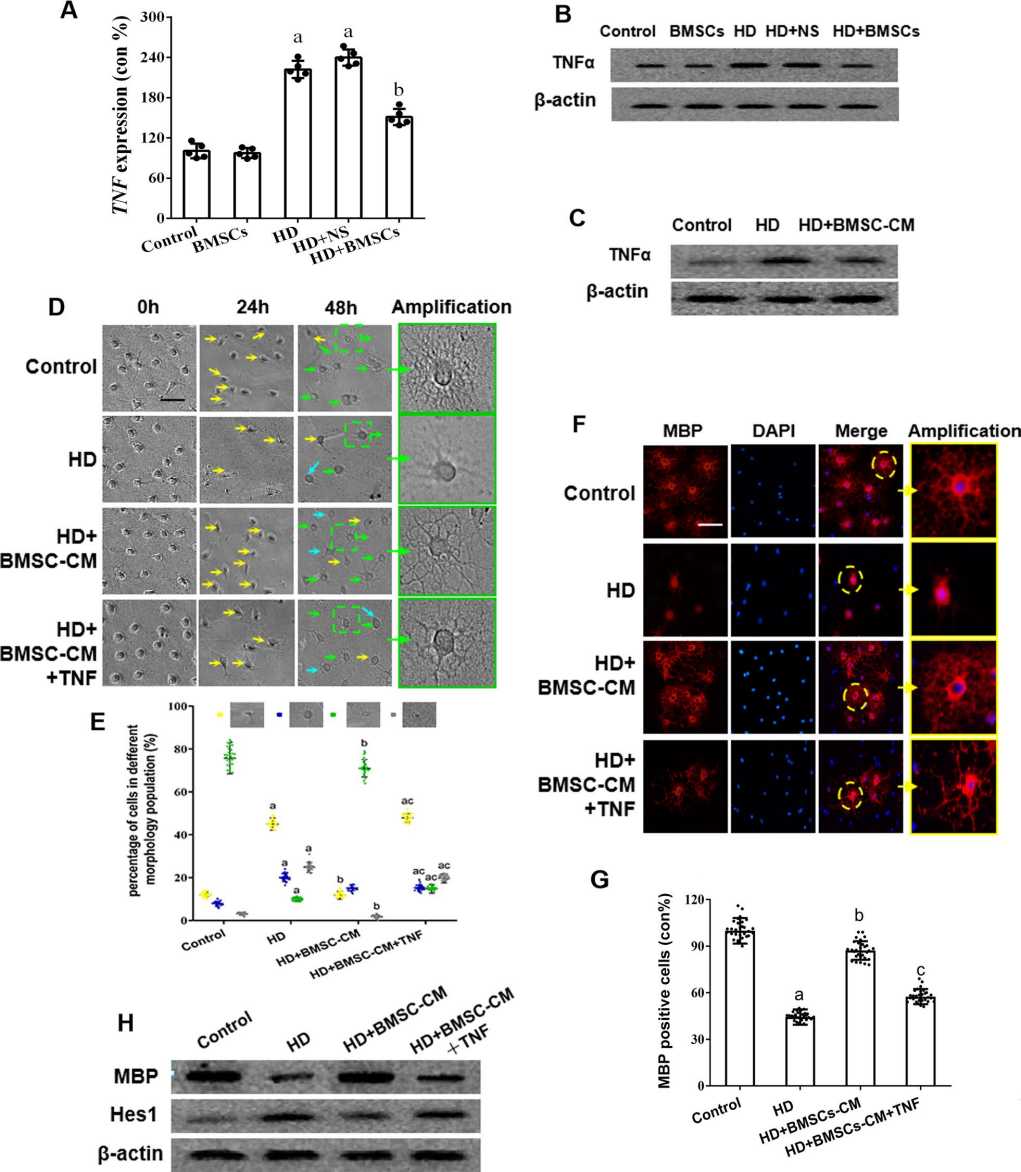
TNFα expression and its relationship with the BMSCs-induced remyelination in HD-exposed rats and OPCs via Hes1 regulation. **a, b** qRT-PCR and western blot results showing TNFα mRNA and protein levels in the spinal cords of HD/BMSCs-treated rats. **c** Western blot results showing TNFα level in HD/BMSC-CM-treated OPCs. **d** Representative phase images of HD/BMSC-CM-treated OPCs ± TNFα treatment. Yellow arrows represent OPCs, cyan arrows represent immature OLs, and green arrows represent mature OLs. Scale bar, 50 μm. **e** Quantification of OPCs, immature OLs, mature OLs and death cell. **f** Representative images showing MBP staining in HD/BMSC-CM-treated OPCs ± TNFα. Scale bar, 50 μm. **g** Quantification of MBP^+^ cells. **h** Western blot results showing MBP and Hes1 expression in HD/BMSC-CM-treated OPCs ± TNFα. Quantified data are shown as mean ± SD. a: Compared with control group, *p* < 0.05; b: compared with HD group, *p* < 0.05

Some studies provided another clue that Hes1 expression could also be modulated by the inflammatory cytokine TNFα [34–36]. To clarify whether TNFα plays a role in BMSCs-induced remyelination, experiments were carried out to compare the mRNA and protein levels of TNFα among different treatment groups in our animal and cell culture models. As a result, RT-PCR (Fig. 5a) and western blot (Fig. 5b) showed that HD intoxication increased TNFα production in rat spinal cord and BMSC transplantation, in turn, attenuated the increase of TNFα. The changes of TNFα protein expression in in vitro OPCs followed the same pattern (Fig. 5c). Therefore, it seemed that TNFα participated in the regulation of oligodendrogenesis in our BMSC-OPC model.

The regulatory role of TNFα was studied by challenging the cultured OPCs in the presence or absence of HD/BMSC-CM with recombinant rat TNFα (10 ng/mL). Using morphological analysis to distinguish oligodendrocyte subpopulations, while BMSCs treatment was found to increase the proportion of mature OLs, supplementation with TNFα to the BMSC-CM-treated cells markedly blocked the increase of OLs (Fig. 5d, e). Similarly, measurement of myelin sheath marker MBP using immunofluorescence (Fig. 5f, g) or western blot (Fig. 5h) identified increased MBP expression upon BMSC-CM treatment and yet no such increase after cells being co-treated with TNFα. In addition, western blot experiment also showed that Hes1, as the negative regulator of oligodendrocyte differentiation, was increased by HD, decreased by BMSC-CM and re-increased by TNFα supplement (Fig. 5i). Together, our results suggested that BMSCs might repress Hes1 expression and promote oligodendrogenesis via inhibiting TNFα production in OPCs.

### Hes1 is an indirect target of TNFα via RelB in BMSCs-regulated oligodendrogenesis

**Fig. 6:**
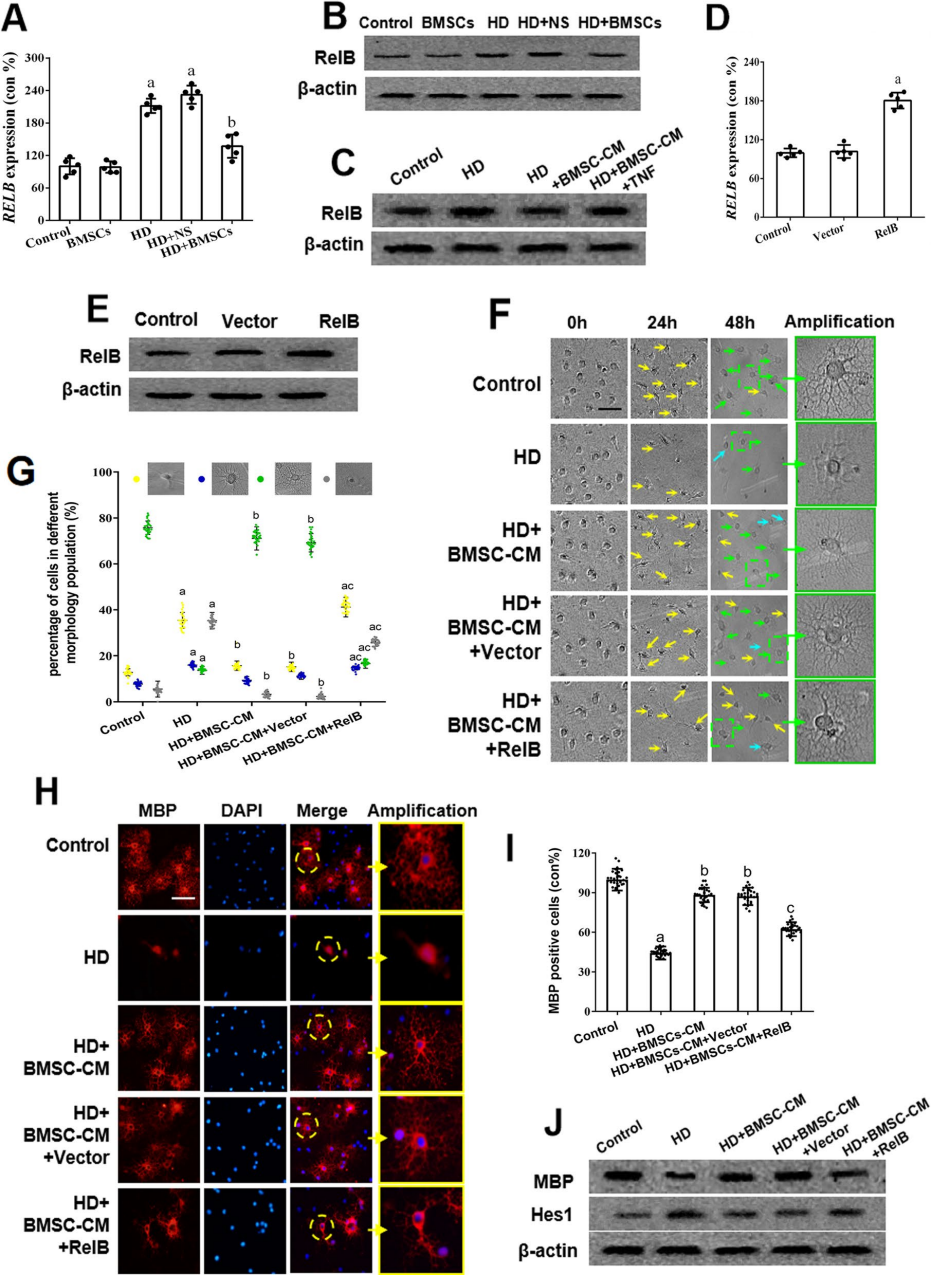
RelB expression and its relationship with the BMSCs-induced remyelination in HD-exposed rats and OPCs via Hes1 regulation. **a, b** qRT-PCR and western blot results showing RelB mRNA and protein levels in the spinal cords of HD/BMSCs-treated rats. **c** Western blot showing RelB expression in OPCs treated with HD/HD + BMSC-CM/HD + BMSC-CM + TNFα. **d, e** qRT-PCR and Western blot showing overexpression of RelB in OPCs. **f** Representative phase images of vector-transfected OPCs or RelB-overexpressing OPCs treated with HD/HD + BMSC-CM. Yellow arrows represent OPCs, cyan arrows represent immature OLs, and green arrows represent mature OLs. Scale bar, 50 μm. **g** Quantification of OPCs, immature OLs, mature OLs and death cell. **h** Representative images showing MBP staining in vector-transfected OPCs or RelB-overexpressing OPCs receiving HD/HD + BMSC-CM treatment. Scale bar, 50 μm. **i** Quantification of MBP^+^ cells. **j, k** Western blot results showing MBP and Hes1 expression in vector-transfected OPCs or RelB-overexpressing OPCs in response to aforementioned treatment. Quantified data are shown as mean ± SD. a: Compared with control group, *p* < 0.05; b: compared with HD group, *p* < 0.05

Since both Hes1 and TNFα seemed to play negative regulatory roles in OPC differentiation (Figs. 3h–l, 5D–H) and both were repressed by BMSC grafting (Figs. 3A–E, 5A–C), we questioned whether these factors could be biologically connected. Some studies suggested that TNFα increased expression of the non-canonical NF-κB protein RelB, which in turn potentiated *Hes1* expression by binding to and promoting nuclear translocation of NICD onto the *Hes1* promoter [35–38]. To examine this potential interconnection, the mRNA and protein levels of RelB were assessed using real time PCR and western blot. Figure 6a, b showed that in rat spinal cords, the RelB expression was increased by HD intoxication and decreased by BMSC treatment. Figure 6c shows that the change of RelB expression also occurred in cultured OPCs receiving BMSC-CM treatment. In addition, the RelB expression was tuned up when the BMSC-CM-treated OPCs were added with recombinant TNFα (Fig. 6c, the last lane).

To further prove that RelB participated in BMSCs-induced remyelination, RelB gene was overexpressed in OPCs (RelB^+^ OPCs) and overexpression status was evidenced (Fig. 6d, e). Investigations were carried out to evaluate the effect of RelB overexpression on BMSC-promoted oligodendrocyte differentiation after HD treatment using in vitro cultured OPCs/RelB^+^ OPCs that were treated with BMSC-CM. As shown in Fig. 6f, g, BMSC-CM failed to increase the number of mature OLs under the condition of RelB overexpression. Similarly, MBP expression assessed by immunofluorescence and western blot was increased in BMSC-CM-treated, HD-exposed OPCs, but not in RelB^+^ OPCs (Fig. 6h–j). The finding was further supported by finding that the decreased level of Hes1 in BMSC-CM-treated OPCs was recovered by RelB overexpression (Fig. 6j). Collectively, our results suggested that BMSCs regulated Hes1 expression and promoted oligodendrogenesis in HD-intoxicated OPCs via TNFα/RelB signaling mediators.

### Inhibition of TNFα production is related with NGF secretion from BMSCs

**Fig. 7:**
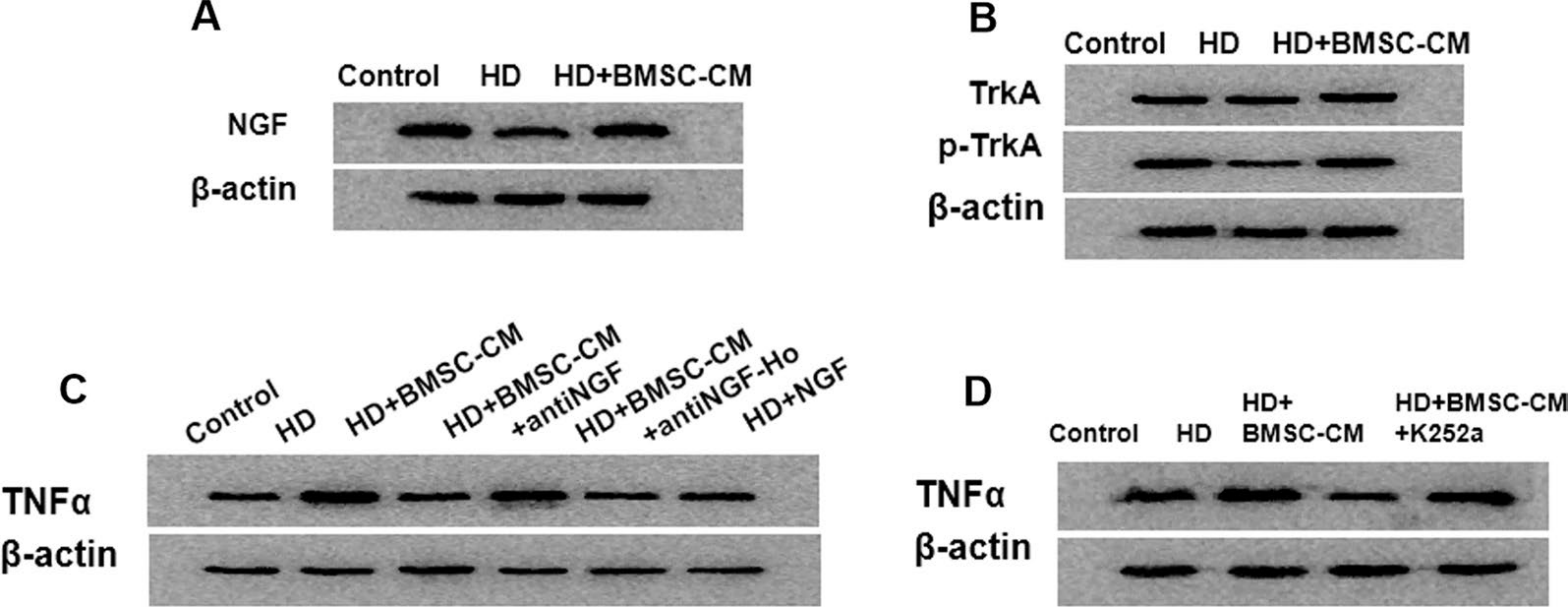
NGF and its relationship with TNFα expression in HD/BMSC-CM-treated OPCs. **a, b** Western blot results showing the level of NGF, p-TrKA and TrKA in HD/HD + BMSC-CM-treated OPCs. **c** Western blot results showing the level of TNFα in HD/HD + BMSCs-treated OPCs receiving additional interference with NGF neutralizing antibody (anti-NGF), negative control antibody (anti-NGF-Ho) or recombinant NGF. **d** Western blot results showing the level of TNFα in HD/HD + BMSCs-treated OPCs without or with TrkA inhibitor K252a treatment. a: Compared with control group, *p* < 0.05; b: compared with HD group, *p* < 0.05; c: compared with HD + BMSC-CM group, *p* < 0.05

Nerve growth factor (NGF), as a secretory neutrophin functioning in a paracrine manner, could be a crucial factor involved in BMSCs’ protective effect against HD-induced damage. Several studies by us [24, 28, 39] and others [40, 41] have shown that the remyelination-promoting effect of BMSCs appeared to be closely related with NGF. Therefore, we further tested whether there be any potential linkage between NGF secretion and TNFα production. Using in vitro cultured OPCs, it was found that the NGF expression (Fig. 7a) and NGF signal activation (represented by p-TrkA) (Fig. 7b) were significantly elevated in response to BMSC-CM treatment. Next, the impact of NGF on TNFα production was evaluated using NGF-neutralizing antibody or recombinant NGF in the cultured OPCs. As shown in Fig. 7c, co-treatment using anti-NGF together with BMSC-CM remarkably abolished the effect of BMSC-CM to repress TNFα production (lane 3 vs lane 4), whereas supplementation with NGF by itself decreased TNFα expression in a similar way that BMSC-CM did (lane 2 vs lane 3 vs lane 6). Whether NGF directly regulates TNFα production was further examined using k252a, an inhibitor of TrkA receptor, that blocks NGF signaling pathway. As shown in Fig. 7d, BMSC-CM decreased TNFα production in OPCs, but this effect was significantly blocked by the addition of K252a. Together, our results suggested that the regulatory role of BMSCs on TNFα production is closely correlated with the secretory neurotrophin factor NGF from BMSCs.

## Discussion

Chronic exposure to N-Hexane, mainly through inhalation, by blue-collar workers in industries with heavy use of adhesives, paint cleansers, solvents in polymer synthetization, oil extraction and rubber processing, is an occupational health problem mainly for developing countries. Prolonged or repetitive N-hexane intoxication typically causes polyneuropathy with axon degeneration and segmental demyelination in the peripheral nerves. The PNS symptoms are usually self-limited after discontinuation of exposure. Alternatively, some studies revealed that hexane intoxication may also induce neuronal damage in CNS, which may persist after PNS symptoms resolved [4, 7, 8]. Another scenario of HD intoxication is recreational abuse such as glue sniffing, which may cause high-dose hexane-associated neurotoxicity and be more likely to cause lesions in CNS [42, 43].

In preclinical studies using experiment animals, high-dose HD intoxication was shown to cause more intensive neurological deficits including demyelinating damage in the spinal cord, which has been employed to evaluate the therapeutical efficacies of various reagents for demyelinating conditions in CNS [9, 44]. In CNS, the repair of damaged myelin sheath is driven by OPCs, acting as glial stem cells, to differentiate into mature OLs to remyelinate the lesioned nerve [45]. However, self-repair driven by OPCs is usually limited and demyelinating conditions in CNS generally require specialized medical interventions. The ideal therapeutical strategy should be well tolerated, safe, easy to carry out and has good targeting efficacy on OPCs.

Stem cell therapy using BMSCs harvested from patients themselves, cultured in vitro and transplanted back to the same individual to achieve certain therapeutical goals has been regarded as a promising approach for demyelinating diseases [46]. BMSCs have autologous HLA compatibility, are easy to obtain and expand, and could be administered via intravenous injection. In our previous study, BMSC transplantation has been found to significantly recover motor function in HD-intoxicated rats [29]. The present study continued our research interest by exploring the responsible molecular mechanism underlying BMSCs’ therapeutical efficacy for spinal cord demyelination induced by HD intoxication using in vivo and in vitro methods. It was evidenced that BMSCs significantly promoted demyelinated nerve fibers to remyelinate over a time course of 5 weeks (recovery period). BMSCs-associated remyelination featured with increased number of mature oligodendrocytes and increased MBP expression. These results are consistent with findings by other research groups. For example, Agrelo et al. reported that certain soluble factors secreted by BMSCs robustly primed the oligodendrogenesis from cultured OPCs and neural stem cells [47]; and the existence of BMSCs was necessary for OPCs grafted in situ into hippocampal slice cultures to differentiate into OLs in vivo [16]. El-Akabawy et al. reported that intravenously injected bone marrow-derived MSCs could promote migration, engraftment and remyelination in the cuprizone model of multiple sclerosis [48].

Our results revealed that BMSCs have a negative regulatory effect on Hes1, likely through a Notch-independent mechanism. Belonging to Hes (hairy and enhancer split) family, Hes1 is a critical gene modulating neurogenesis. Hes1 gene encodes a basic helix-loop-helix (bHLH) transcriptional repressor that antagonizes with positive bHLH transcription factors [49]. Hes1 overexpression has been found to inhibit cell differentiation and promote precursor cells to proliferative [50]. Transgenic Hes1 knockout mice displayed a characteristic phenotype of cerebellar hypoplasia caused by defected neuronal proliferation along with unrestrained differentiation [49]. The present work showed that Hes1 expression was significantly upregulated after HD treatment. This was also supported by our microarray data profiling gene expression in rat spinal cords after HD intoxication (unpublished yet). In comparison, Hes1 expression was found to be significantly repressed during the process of BMSCs-induced remyelination and Hes1 overexpression in OPCs could attenuate oligodendrogenesis. Together, our results suggested that Hes1 is a crucial regulatory target of BMSCs.

Next, the present study examined by what mechanism(s) BMSCs regulate Hes1, a downstream transcriptional target of Notch signaling pathway [33]. Notch1 is a transmembrane receptor and activated by ligands including Jagged1. Notch1 is then cleaved and its intracellular domain NICD, as the activated form, is released, translocates to nucleus, binds to co-factor RBPJ and transcribes Hes1 promoter [22, 51]. Our work compared the expression of Jagged1, Notch1, NICD (activated Notch1) and RBPJ between HD-treated and HD + BMSCs or BMSC-CM treated groups, and yet, no significant difference was found in both in vivo and in vitro models. Our finding is not too surprising, giving the fact that the exact regulatory role of Notch during remyelination remains controversial. While Zhang et al. reported that Notch1 regulated OPCs differentiation using an OPC-specific Notch1-deletion mouse model and lysolecithin microinjection-induced focal demyelination in corpus callosum [51], Stidworthy et al. found that Notch1 ablation in OPCs yielded no significant difference in oligodendrogenesis between transgenic mice and wild-type littermates in response to cuprizone-induced demyelinating damage in corpus callosum [52]. Eykens et al. reported that the ablation of Notch1 in OPCs failed to interfere with oligodendrocyte differentiation using an amyotrophic lateral sclerosis mouse model [53]. It is perhaps reasonable to speculate that the regulatory effect of Notch1 could depend on more-than-one factors, such as animal species (rat vs mouse), the type of demyelinating toxin (lysolecithin vs cuprizone vs N-hexane), the specific nervous tissue (corpus callosum vs spinal cord) and the other microenvironmental factors such as the copresence of different inflammatory cytokines.

The present study suggested that BMSCs regulated Hes1 expression through repression of the proinflammatory cytokine TNFα and non-canonical NF-κB subunit RelB. Overexpression of TNFα or RELB reversed the negative regulation by BMSCs on Hes1 expression. This finding is interesting and yet not unexpected. Indeed, some studies suggested that BMSCs cross-talked with immunomodulatory factors to gain full potentiation of their remyelinating ability. A previous study observed that when the peripheral immune response was experimentally blocked in rat, BMSCs lost their ability to promote neural function [54]. Neurological studies revealed TNFα have important roles in embryonic and adult neurogenesis [55]. Indeed, some studies proposed that the regulation of Hes1 relied on the conjunctive work of both Notch1 and TNFα [34–36]. These studies revealed that TNFα increased RelB expression, promoted RelB binding to NICD and the RelB-NICD cofactors translocated to nucleus to drive Hes1 expression [36–38].

The work also attempted to explain by which mechanism BMSC regulated TNFα expression and associated these phenomena with the neurotrophic factor NGF. NGF is a relevantly well-characterized neurokine secreted by BMSC. NGF binds to its receptor TrkA and drives neuron survival and neurite outgrowth via PI3K/Akt and Ras/MAPK signaling, respectively. Investigations by other groups also demonstrated that NGF could dampen inflammatory response via downregulating proinflammatory cytokines including TNFα [56, 57]. Our study found that NGF inhibited TNFα expression and such inhibitory effect was abolished by NGF neutralizing antibody or TrkA inhibitor. These results imply that NGF secreted by BMSCs inhibited TNFα/RelB pathway and induced remyelination, in consistence with the results of other groups [56, 58].

## Conclusions

In summary, the present study demonstrated that BMSCs transplantation promoted remyelination in nervous tissue (rat spinal cord) after demyelinating damage caused by *N*-hexane, a hazardous material used in industry. Mechanistically, our in vitro results suggested that BMSCs could release NGF to inhibit TNFα production and TNFα/RelB pathway, thereby reducing the level of Hes1, a transcription factor driving OPCs away from differentiation, in a Notch1-independent manner. Our in vivo experiments also observed the changed expression of Hes1, TNFα and RelB molecules in response to BMSCs transplantation. Although BMSC transplantation may have certain limitations, it represents a promising next-generation approach to manage demyelinating neurodegenerative diseases. The findings by us in the present work and earlier published studies might provide certain novel insights and a model to compare with to other investigators conducting similar researches exploring the effectiveness and mechanisms of BMSCs to treat demyelinating conditions (using different models). Ongoing investigations include evaluation of the acellular exosome components derived from BMSCs to treat HD-intoxication and further analysis of the core signaling molecules associated with BMSCs’ action.

## Supplementary Information

**Additional file 1: Figure 1**. The quantification results of western blots. S1.A: Quantification of WB bands of Fig. 2c; S1.B: Quantification of WB bands of Fig. 2f; S1.C: Quantification of WB bands of Fig. 3b; S1.D: Quantification of WB bands of Fig. 3e; S1.E: Quantification of WB bands of Fig. 3g; S1.F: Quantification of WB bands of Fig. 3l; S1.G-J: Quantification of WB bands of Fig. 4e; S1.K-N: Quantification of WB bands of Fig. 4j; S1.O: Quantification of WB bands of Fig. 5b; S1.P: Quantification of WB bands of Fig. 5c; S1.Q-R: Quantification of WB bands of Fig. 5h; S1.S: Quantification of WB bands of Fig. 6b; S1.T: Quantification of WB bands of Fig. 6c; S1.U: Quantification of WB bands of Fig. 6e; S1.V-W: Quantification of WB bands of Fig. 6j; S1.X: Quantification of WB bands of Fig. 7a; S1.Y: Quantification of WB bands of Fig. 7b; S1.Z: Quantification of WB bands of Fig. 7c; S1.Z1: Quantification of WB bands of Fig. 7d

## Data Availability

All data generated or analyzed during this study are included in this published article. Further requests are welcomed and please contact the corresponding authors.

## Abbreviations

ANOVA: One-way analysis of variance
BMSC: Bone marrow mesenchymal stem cells
BMSC-CM: BMSC-derived conditioned medium
HD: Hexanedione
LFB: Luxol-fast blue
NG2: Neural/Glial Antigen 2
NGF: Nerve growth factor
NS: Normal saline
OL: Oligodendrocyte
OPC: Oligodendrocyte progenitor cells
MBP: Myelin basic protein
SD: Sprague Dawley
STZ: Streptozotocin
TNFα: Tumor necrosis factor alpha
TrkA: Tyrosine kinase A
