# Supplementary figures and images for: Bone marrow mesenchymal stem cells promote remyelination in spinal cord by driving oligodendrocyte progenitor cell differentiation via TNFα/RelB-Hes1 pathway: a rat model study of 2,5-hexanedione-induced neurotoxicity
Source: Stem Cell Res Ther. 2021 Aug 4;12:436. doi: 10.1186/s13287-021-02518-z (PMC8336089; doi:10.1186/s13287-021-02518-z)

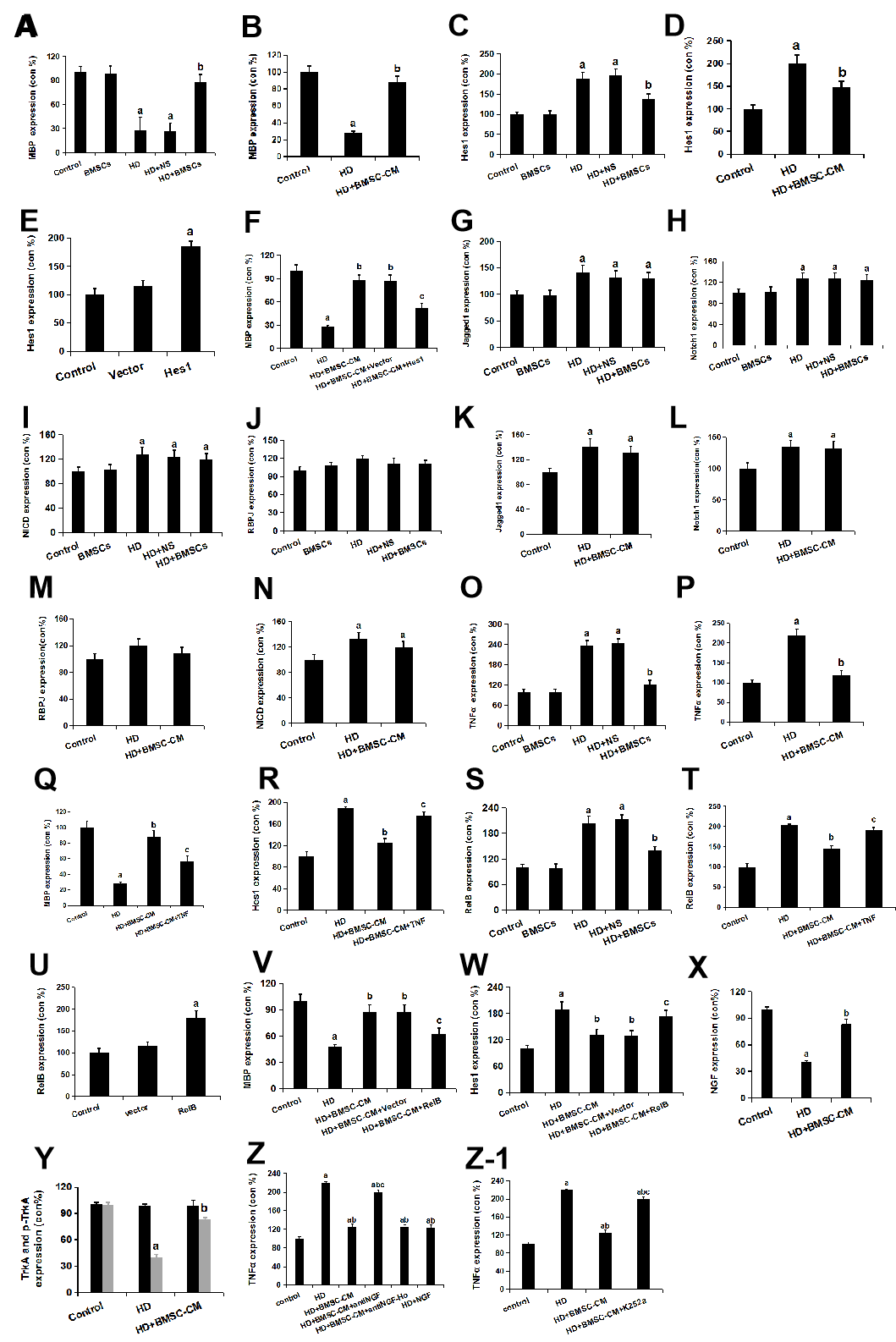

Supplement: Supplementary file 1 — Additional file 1: Figure 1. The quantification results of western blots. S1.A: Quantification of WB bands of Fig. 2c; S1.B: Quantification of WB bands of Fig. 2f; S1.C: Quantification of WB bands of Fig. 3b; S1.D: Quantification of WB bands of Fig. 3e; S1.E: Quantification of WB bands of Fig. 3g; S1.F: Quantification of WB bands of Fig. 3l; S1.G-J: Quantification of WB bands of Fig. 4e; S1.K-N: Quantification of WB bands of Fig. 4j; S1.O: Quantification of WB bands of Fig. 5b; S1.P: Quantification of WB bands of Fig. 5c; S1.Q-R: Quantification of WB bands of Fig. 5h; S1.S: Quantification of WB bands of Fig. 6b; S1.T: Quantification of WB bands of Fig. 6c; S1.U: Quantification of WB bands of Fig. 6e; S1.V-W: Quantification of WB bands of Fig. 6j; S1.X: Quantification of WB bands of Fig. 7a; S1.Y: Quantification of WB bands of Fig. 7b; S1.Z: Quantification of WB bands of Fig. 7c; S1.Z1: Quantification of WB bands of Fig. 7d [file 13287_2021_2518_MOESM1_ESM.tif]
